# Comparative Evaluation of Pressurized Liquid and Conventional Solvent Extraction of Agro-Industrial By-Products: Phenolic and Flavonoid Contents, Antioxidant Capacity and Cytotoxicity Assessment

**DOI:** 10.3390/foods15203636

**Published:** 2026-10-13

**Authors:** Maria Luigia di Corcia, Cristina Luceri, Elisabetta Bigagli, Amalia Conte, Matteo Alessandro Del Nobile

**Affiliations:** 1 Department of Humanistic Studies, Letters, Cultural Heritage, Educational Sciences, University of Foggia, Via Arpi, 176, 71121 Foggia, Italy; maria.dicorcia@unifg.it; 2 Department of Neuroscience, Psychology, Drug Research and Child Health (NEUROFARBA), Section of Pharmacology and Toxicology, University of Florence, Viale Pieraccini 6, 50139 Florence, Italy; cristina.luceri@unifi.it (C.L.); elisabetta.bigagli@unifi.it (E.B.); 3 Department of Economics, Management and Territory, University of Foggia, Via A. da Zara, 11, 71122 Foggia, Italy; matteo.delnobile@unifg.it

**Keywords:** by-products, solvent extraction, pressurized liquid extraction, sustainability, antioxidant activity, cytotoxicity

## Abstract

In the present study, extracts obtained from four agro-industrial by-products, namely pomegranate peels (*Punica granatum* L.), turnip tops (*Brassica rapa* L.) residues, prickly pear (*Opuntia ficus-indica* L.) peels, and artichoke (*Cynara cardunculus* var. *scolymus*) stems and leaves, were produced by conventional solvent extraction (acidified methanol–water, 80:20, *v*/*v*) and water-based pressurized liquid extraction (PLE). The extracts were comparatively evaluated for total phenolic and flavonoid contents, chemical antioxidant capacity by ABTS and FRAP assays, and cytotoxicity toward Caco-2 cells. Conventional solvent extraction generally resulted in higher Folin–Ciocalteu-estimated total phenolic contents and ABTS activity, whereas flavonoid contents were comparable between the two extraction procedures. Pomegranate peel exhibited the highest values among the investigated matrices, with total phenolic contents of 141 and 193 mg GAE/g dry matter for PLE and conventional extraction, respectively and flavonoid content of approximately 100 mg QE/g dry matter for both procedures. Consistently, pomegranate peel showed the highest ABTS and FRAP responses, reaching 492 mg Trolox/g dry matter and 6032 μmoL Fe (II)/g dry matter for PLE and 618 mg Trolox/g dry matter and 8015 μmoL Fe (II)/g dry matter for conventional extraction, respectively. In contrast, PLE resulted in higher FRAP responses for all three remaining matrices, namely turnip tops, prickly pear, and artichoke by-products, with values approaching twice those obtained by conventional extraction for turnip tops and artichoke. Among these matrices, turnip tops showed the highest FRAP response, followed by prickly pear and artichoke, whereas prickly pear exhibited higher ABTS activity than turnip tops and artichoke. Overall, the non-pomegranate matrices showed substantially lower phenolic and flavonoid contents and chemical antioxidant capacity than pomegranate peel. Extracts generally showed low cytotoxic toward Caco-2 cells under the experimental conditions tested, with cell viability remaining close to or above 80% across the tested concentrations, although some matrix- and concentration-dependent effects were observed. These results demonstrate that the relative performance of conventional solvent extraction and PLE depended on both the plant matrix and the analytical endpoint: conventional extraction produced higher phenolic and ABTS responses for pomegranate peel, whereas water-based PLE yielded higher FRAP responses for the other matrices while providing comparable flavonoid contents. Thus, the findings highlight the importance of considering both matrix characteristics and the analytical endpoint when comparing extraction procedures for agro-industrial by-product valorization. The study provides a preliminary in vitro characterization of the investigated extracts, while further phytochemical and biological investigations are required to establish their potential for food-related applications.

## 1. Introduction

Plant-derived by-products are increasingly recognized as valuable sources of bioactive compounds, offering a sustainable approach to the production of functional extracts. These materials, often considered waste in agricultural and food processing chains, can be transformed into high-value products with demonstrated benefits for human health [1,2]. Numerous studies have highlighted their potential not only to promote physiological well-being, through antioxidant, anti-inflammatory, and metabolic effects, but also to exert targeted actions against toxic cells [3,4]. Although the scientific literature already provides substantial evidence supporting these applications, further research is still needed. This is largely due to the vast diversity of plant raw materials and their by-products available and the wide range of extraction techniques, which can significantly influence the composition, efficacy, and safety of the resulting extracts [5,6,7,8].

Notably, most existing studies have focused on conventional solvent-based extractions, while comparatively limited attention has been given to extracts obtained through pressurized liquid extraction (PLE). This technique offers several advantages, including reduced extraction time, lower solvent consumption, and improved extraction efficiency using elevated temperature and pressure [9,10]. Importantly, PLE also allows the use of water as a co-solvent, making it a more environmentally friendly and potentially safer alternative for obtaining bioactive compounds [11]. Despite these benefits, further investigation is required to fully explore its potential and to better understand how extraction parameters affect the biological activity of the resulting extracts. Although numerous studies have investigated the phenolic content and antioxidant properties of individual agro-industrial by-products, direct comparisons of different extraction strategies across chemically distinct matrices under a common experimental framework remain comparatively limited. Extraction performance may depend not only on the characteristics of the plant matrix and the extraction technology, but also on the analytical endpoint used to characterize the resulting extracts.

The selection of specific plant by-products such as pomegranate peels, turnip tops, prickly pear, and artichokes stems and leaves is well justified by their rich phytochemical profiles and widespread availability as agro-industrial waste. Pomegranate peels are known to contain high levels of polyphenols, including ellagitannins and flavonoids, with strong antioxidant and antiproliferative properties [12,13]. Although most studies use whole fruit, some findings are also available on extracts from fruit by-products, as peels and seeds [14,15]. Methanol extract of pericarp of pomegranate has been shown to exert antiproliferative effects on human breast cancer cells in vitro, demonstrating the potential for prevention of estrogenic-dependent breast cancers with beneficial effects in other hormone-dependent tissues [16]. Low doses of methanolic extracts prepared from pomegranate peel exerted incredibly significant alteration in tongue cancer cell proliferation, inducing apoptosis and inhibition of cancer cell migration [17]. Turnip tops and broccoli by-products, belonging to the Brassicaceae family, are particularly rich in glucosinolates and their bioactive derivatives, such as isothiocyanates, which have been associated with detoxifying and chemo-preventive activities [18,19]. Various parts of the Opuntia plant, including the fruits, stems/cladodes, and roots, have demonstrated cytotoxic effects against malignant cell lines in numerous studies [20]. Prickly pear residues are an important source of betalains, vitamins, and phenolic compounds with notable antioxidant and cytoprotective effects [21,22]. Likewise, artichoke stems and outer leaves, which constitute a substantial fraction of processing waste, are rich in bioactive molecules such as chlorogenic acid, caffeoylquinic acids, cynarin, and flavonoids. These compounds have been widely associated with antioxidant, hepatoprotective, anti-inflammatory, and antiproliferative activities [23,24].

In this context, the valorization of these materials can contribute to waste reduction and circular economy strategies while providing sources of compounds of potential interest for further applications. Therefore, a comparative assessment of conventional solvent extraction and pressurized liquid extraction (PLE) applied to different agro-industrial by-products can provide useful information on the extent to which the extraction response is matrix- and assay-dependent, supporting a more informed evaluation of extraction strategies for by-product valorization. To this aim, the present study compared extracts obtained from selected plant by-products using PLE with water as the extraction solvent with extracts produced by conventional solvent extraction using an acidified methanol–water mixture. The study evaluated total phenolic and flavonoid contents, chemical antioxidant capacity by ABTS and FRAP assays, and the effects of the extracts on Caco-2 cell viability under the tested conditions. Particular attention was given to the extent to which the two extraction procedures produced different responses depending on the plant matrix and analytical endpoint. The study was intended to provide a preliminary in vitro characterization of the extracts and to contribute to the evaluation of extraction approaches for the valorization of agro-industrial by-products, while identifying aspects requiring further investigation before potential food-related applications can be considered.

## 2. Material and Methods

### 2.1. Raw Materials

Turnip tops (*Brassica rapa* L. subsp. sylvestris), artichokes (*Cynara cardunculus* var. scolymus), prickly pears (*Opuntia ficus-indica*) and pomegranates (*Punica granatum* L.) were purchased as whole, commercially available products from local retailers in the Apulia Region (Italy). The fractions investigated as by-products were obtained by the authors during sample preparation. Specifically, pomegranate and prickly pear peels were manually separated from the whole fruits; artichoke leaves and stems were separated from the edible portions; and the harder, non-edible portions of turnip tops, which are normally removed during preparation, were collected. These fractions were considered by-products because they represent secondary materials separated from the main food product during preparation and are potentially recoverable for further valorization. For each plant material, a single batch was collected and used for both extraction procedures and subsequent analyses. No formal botanical authentication by a taxonomist was performed, and voucher specimens were not deposited in a recognized herbarium. Upon arrival at the laboratory, all whole plant materials were stored at 4 °C until further processing. The samples were thoroughly washed under running tap water to remove surface impurities and subsequently sanitized by immersion in chlorinated water solution (20 mL/L) for 5 min. The samples were then rinsed with distilled water and air-dried at room temperature. After washing and sanitization, the fractions considered as by-products were manually separated from the whole plant materials. The resulting by-product fractions were then processed for dehydration. Prior to dehydration, artichoke and turnip tops by-products were manually cut into small pieces. All the by-products were dehydrated using a conventional hot-air dryer (PF-SICCO80PRO, SICCOTECH, Campobasso, Italy) at 60 °C and 5% relative humidity, following the procedure described by Le Rose et al. [25]. Drying may cause the degradation, loss, or transformation of thermolabile and oxidation-sensitive compounds and may therefore have influenced the absolute values of phenolic content and antioxidant capacity measured in the extracts. However, the same dehydration procedure was applied to each batch before the dried material was divided between the two extraction procedures. Thus, drying represents a common pre-treatment rather than a factor differentiating PLE from conventional solvent extraction, although the possible effect on the absolute recovery of sensitive compounds should be considered when interpreting the results. After dehydration, all samples were ground using a laboratory mill (16/BV, Beccaria S.r.l., Cuneo, Italy) to obtain a fine powder with a particle size of <500 μm. The powders were vacuum sealed in plastic bags and stored at 4 °C until further use.

### 2.2. Extraction Process

The powders obtained from the four agro-industrial by-products (pomegranate peels, prickly pear peels, turnip tops, and artichoke bracts and leaves) were subjected to two extraction procedures: conventional solvent extraction and Pressurized Liquid Extraction (PLE). For conventional solvent extraction, 2 g of each dried by-product were mixed with 20 mL of acidified methanol (80% methanol in water containing 1% HCl, *v*/*v*). The mixtures were shaken in the dark at room temperature for 2 h at 3000 rpm using an orbital shaker (HS 260 BASIC, IKA, Staufen, Germany), followed by centrifugation at 4 °C for 10 min at 10,000 rpm (5804R, Eppendorf, Milan, Italy). The supernatants were collected and filtered through 0.45 μm PTFE membrane filters before analytical determinations. All extractions were performed in triplicate, and the resulting extracts were appropriately diluted, when necessary, prior to analysis, according to the method described by Lordi et al. [26].

PLE was performed using a Pressurized Liquid Extraction system (LabService Analytica S.r.l., Anzola dell’Emilia, Bologna, Italy) according to the method described by Marinelli et al. [27], with the modifications detailed below. For sample preparation, 25 g of dried by-product were mixed with 25 g of Ottawa sand and 10 g of diatomaceous earth to improve solvent percolation through the sample matrix, corresponding to a by-product sand earth ratio of 1:1:0.4 (*w*/*w*/*w*). The mixture was pre-hydrated with 25 mL of distilled water for all matrices, except for turnip tops, for which 50 mL of distilled water was used. After homogenization, 15 g of the prepared mixture were loaded into a 35 mL extraction cell lined with filter paper. Distilled water was used as the extraction solvent, with 100 mL used in each extraction cycle. Six extraction cycles were performed for each by-product, corresponding to a total extraction solvent volume of approximately 600 mL per matrix. For pomegranate and prickly pear peels, extraction was performed under dynamic conditions. Each cycle included a 5 min heating step at 60 °C followed by a dynamic extraction phase at 70 °C lasting 20 min, with a total cycle duration of approximately 45 min. For artichoke bracts and leaves and turnip tops, extraction was performed under static conditions. Each cycle included a 5 min heating step at 60 °C followed by a static extraction step at 70 °C lasting 20 min, with a total cycle duration of approximately 30 min. The extraction pressure was set at 1500 psi. At the end of each extraction cycle, the extraction cell was rinsed with fresh solvent and purged with nitrogen for 2 min. The aqueous extracts obtained from the six cycles were collected and combined for each by-product, resulting in approximately 600 mL of combined extract before drying. The combined extracts were dried in a forced-air oven (BINDER GmbH, Tuttlingen, Germany) at 40 °C until constant weight. The dried extracts were then ground to obtain a homogeneous powder and stored at −20 °C until further analysis. The masses of the resulting dry extracts were 7.36 g for pomegranate peel, 6.10 g for prickly pear peel, 2.57 g for artichoke by-products, and 5.46 g for turnip tops. For the analytical determinations, 2 g of each dried PLE-extract were acidified in methanol (80% methanol in water containing 1% HCl, *v*/*v*) and then centrifuged under the same conditions adopted for the conventional solvent extraction before assessment of their nutritional quality.

### 2.3. Phytochemical and Antioxidant Assays

The following analytical-grade reagents were used throughout the study: Folin–Ciocalteu reagent, gallic acid monohydrate, anhydrous sodium carbonate, methanol, hydrochloric acid, 2,2-azino-bis(3-ethylbenzothiazoline-6-sulfonic acid) diammonium salt (ABTS), potassium persulfate, Trolox (6-hydroxy-2,5,7,8-tetramethylchroman-2-carboxylic acid), aluminum chloride, sodium nitrite, sodium hydroxide, and quercetin. All chemicals were purchased from Sigma-Aldrich (Milan, Italy). Total phenolic content (TPC) was determined using the Folin–Ciocalteu assay according to the method described by Panza et al. [28] and expressed as mg gallic acid equivalents (GAE)/g dry weight (dw), based on a calibration curve ranging from 3.12 to 100 mg/L (R^2^ = 0.999). Since the Folin–Ciocalteu reagent is not specific exclusively to phenolic compounds and can also react with other reducing substances, the values obtained are considered an estimate of the total phenolic content rather than a specific quantification of individual or total phenolic compounds. Total flavonoid content (TFC) was determined using the aluminum chloride colorimetric assay according to the method described by Panza et al. [28] and expressed as mg quercetin equivalents (QE)/g dw, using a calibration curve ranging from 6.25 to 400 mg/L (R^2^ = 0.995). Antioxidant activity was evaluated using the ABTS radical scavenging assay according to the method described by di Corcia et al. [29] and expressed as mg Trolox equivalents (TE)/g dw, based on a calibration curve ranging from 12.5 to 500 mg/L (R^2^ = 0.990). Ferric reducing antioxidant power (FRAP) was determined according to the method described by di Corcia et al. [29] and expressed as μmoL Fe(II) equivalents/g dw, using a calibration curve established between 0.1 and 0.8 mmol/L (R^2^ = 0.993). All measurements were performed in triplicate. All analytical results were expressed on a dry-weight basis, referring to the mass of the dried extract obtained after extraction. Thus, values reported as mg GAE/g dry weight, mg QE/g dry weight, and antioxidant capacity per g dry weight refer to g of dry extract, rather than to the initial dry raw material. This normalization was applied consistently to both PLE and conventional solvent extracts, allowing direct comparison of the analytical responses of the resulting dried extracts.

### 2.4. Extract Cytotoxicity

Caco-2 cells (ATCC, Manassas, VA, USA) were cultured in Dulbecco’s Modified Eagle Medium (DMEM, EuroClone, Milan, Italy) supplemented with 20% heat-inactivated foetal calf serum, 1% L-glutamine, and 1% penicillin/streptomycin, and maintained at 37 °C in a humidified incubator with 5% CO_2_. Cell viability was assessed using the CellTiter 96™ AQueous One Solution Cell Proliferation Assay based on the tetrazolium compound MTS [3-(4,5-dimethylthiazol-2-yl)-5-(3-carboxymethoxyphenyl)-2-(4-sulfophenyl)-2H-tetrazolium] (Promega, Madison, WI, USA). The assay is based on the reduction of MTS by metabolically active cells to a soluble coloured formazan product. The amount of formazan produced, measured spectrophotometrically, is proportional to cellular metabolic activity and provides an indirect measure of cell viability. Briefly, Caco-2 cells were seeded into flat-bottom 96-well tissue culture plates (Corning, Corning, NY, USA) at a density of 1 × 10^4^ cells/well and allowed to adhere for 24 h under the culture conditions described above. The culture medium was then replaced with fresh medium containing the extracts at final concentrations ranging from 25 to 500 µg/mL, and cells were exposed for 72 h. The water-based PLE extracts were dissolved in sterile water, whereas the extracts obtained by conventional solvent extraction were dissolved in DMSO to prepare 100 mg/mL stock solutions. Stock solutions were subsequently diluted in complete culture medium to obtain the final concentrations used for cell treatment (25–500 µg/mL). The final DMSO concentration did not exceed 0.5% (*v*/*v*), corresponding to the highest extract concentration tested. Cells treated with 0.5% DMSO, corresponding to the maximum vehicle concentration used in the experiments, were included as vehicle controls. Cell-free controls containing the extracts and MTS reagent were included to assess potential interference of the extracts with the colorimetric assay. Following treatment, MTS reagent was added to each well, and the plates were incubated for an additional 2 h at 37 °C. Absorbance was measured at 490 nm using a Multilabel Counter 1240 Victor^3^ spectrophotometer (PerkinElmer, Waltham, MA, USA). Cell viability was expressed as a percentage of the untreated control, with values calculated from three independent replicates.

### 2.5. Statistical Analysis

For each by-product, both extraction procedures (conventional solvent extraction and PLE) were considered, resulting in eight experimental groups overall (four by-products × two extraction procedures). The statistical analysis was performed by comparing all eight extracts with one another, to assess differences both between different by-products and between the two extraction procedures. A one-way analysis of variance (HSD of Tukey), with the option of homogeneous groups (*p* < 0.05) was used to determine significant differences among samples. For the MTS assay, data were analysed by one-way ANOVA followed by Dunnett’s post hoc test. A *p* value < 0.05 was considered statistically significant. JMP 18 for Windows (JMP Statistical Discovery LLC, Cary, NC, USA) was used.

## 3. Results and Discussion

### 3.1. Phenolic and Flavonoid Content

Results on polyphenols and flavonoids of tested extracts are reported in Table 1 for both extraction techniques adopted. The total polyphenol content analysis revealed a markedly different behaviour among the investigated plant by-products. Pomegranate peel extracts showed by far the highest concentration of Folin–Ciocalteu-estimated TPC regardless of the extraction technique employed, reaching values of 193.32 ± 0.26 and 141.72 ± 0.4 mg GAE g^−1^ dry matter for the conventional solvent extraction (acidified methanol–water, 80:20 *v*/*v*) and PLE, respectively. These values were substantially higher than those measured in all the other extracts, whose Folin–Ciocalteu-estimated TPC were generally around or below 15 mg GAE g^−1^ dry matter. Such a pronounced difference indicates a greater response in the assay, which may reflect differences in the amount and/or nature of phenolic and other reducing compounds extracted. Literature states that unlike many other fruit and vegetable by-products, pomegranate peels accumulate large amounts of hydrolyzable tannins, particularly ellagitannins such as punicalagins, together with ellagic acid derivatives, gallic acid, and various flavonoids. These compounds are naturally concentrated in the peel tissue, where they contribute to plant defence mechanisms against environmental stressors, pathogens, and oxidative damage [30]. As a result, the peel can contain significantly higher levels of phenolics and bioactive agents than the edible arils [31,32]. However, the present results should be interpreted as Folin–Ciocalteu-estimated TPC, since individual phenolic compounds were not identified or quantified. The higher TPC obtained by conventional extraction for pomegranate peel may be related to differences in the extraction media and operating conditions. Conventional extraction used an acidified methanol–water mixture, whereas PLE employed water as the extraction solvent. Solvent composition is an important factor affecting the extraction response of phenolic compounds from plant matrices, and hydroalcoholic mixtures may provide different extraction efficiencies from water alone because of differences in solvent polarity and compound solubility [32,33,34]. These differences may be particularly relevant for pomegranate peel because of the diverse range of phenolic constituents reported for this matrix. Nevertheless, the present experimental design does not allow us to determine whether the higher TPC obtained with the conventional procedure resulted from preferential recovery of specific phenolic compounds. The extraction conditions associated with PLE may also have contributed to the observed differences. Pressurized water at elevated temperature has physicochemical properties that differ from those of water under conventional conditions and may affect solvent penetration, mass transfer, and compound release from the plant matrix [33,34]. Temperature may also influence the stability of extracted compounds; however, because individual compounds and their possible degradation or transformation were not monitored, such effects cannot be established from the present results. The results indicate that extraction performance was strongly matrix-dependent, with pomegranate peel showing a markedly greater phenolic response than the other by-products under both extraction conditions. The substantially lower TPC values observed for turnip tops, prickly pear residues, and artichoke stems and leaves compared with pomegranate peel may reflect the marked differences in the phenolic characteristics of these matrices. The phenolic constituents of these by-products have been widely reported in the literature, although their abundance and distribution among different classes of compounds may vary considerably depending on the plant material [35,36]. Therefore, the approximately ten-fold or greater difference in TPC between pomegranate peel and the other matrices is consistent with the documented phenolic richness of pomegranate peel [32]. However, because individual compounds were not characterized in the present study, the contribution of specific phenolic classes or compounds to the measured TPC cannot be directly established.

A similar trend to that observed for TPC was found for total flavonoid content (TFC) (Table 1). Among the investigated extracts, pomegranate peel exhibited the highest TFC with values close to 100 mg QE g^−1^ dry matter for both extraction procedures. In contrast, the other by-products generally showed considerably lower flavonoid levels, around or below 10 mg QE g^−1^ dry matter. These findings confirm the outstanding phytochemical richness of pomegranate peel compared with the other matrices analysed [36,37]. However, the present results represent an overall estimate of flavonoid content and do not provide information on the individual flavonoid compounds contributing to this response. Unlike the results obtained for TPC, no statistically significant difference (*p* > 0.05) was observed between the conventional solvent extraction and PLE extracts of pomegranate peel. Thus, under the conditions investigated, the two extraction procedures produced similar TFC responses for this matrix. This result may indicate that the use of pressurized water was sufficient to obtain a flavonoid response comparable to that obtained with the acidified methanol–water mixture. The physicochemical properties of the extraction medium, together with the temperature and pressure applied during PLE, can influence the solubilization and release of compounds from plant matrices [34,38]. Nevertheless, because individual flavonoids were not identified or quantified, the present data do not allow us to determine whether the two extraction procedures recovered similar flavonoid profiles or whether specific compounds contributed differently to the measured TFC. The exceptionally high TFC of pomegranate peel is consistent with previous reports describing the peel as a phenolic-rich plant fraction [39]. The peel is continuously exposed to environmental stresses such as ultraviolet radiation, microbial attack, and oxidative challenges, leading to the accumulation of large amounts of secondary metabolites, that contribute to antioxidant defence and plant protection; however, this aspect cannot be directly evaluated from the present experimental data. Conversely, the much lower TFC observed in turnip tops, prickly pear residues, and artichoke stems and leaves may reflect differences in the abundance and distribution of flavonoid-containing compounds among the investigated matrices. Although these matrices contain bioactive compounds with important biological functions, their phytochemical profiles are often characterized by a greater contribution of other classes of metabolites, such as phenolic acids, glucosinolate derivatives, betalains, or caffeoylquinic acids, rather than flavonoids [40,41,42]. As a result, the flavonoid fraction represents a smaller proportion of their total bioactive content, explaining the values that were generally more than one order of magnitude lower than those measured in pomegranate peel extracts. Since individual phytochemicals were not characterized, the contribution of specific flavonoid classes or compounds to these differences cannot be established.

### 3.2. Extract Antioxidant Capacity

The antioxidant capacity evaluated by the ABTS and FRAP assay is reported in Table 2. ABTS results showed a pattern largely consistent with the results obtained for TPC and TFC. Pomegranate peel extracts exhibited the highest radical scavenging activity among all the investigated samples, with significantly greater values observed for the conventional solvent extraction compared with PLE. The markedly higher antioxidant capacity of pomegranate peel is not surprising considering its exceptionally high concentration of Folin–Ciocalteu-estimated TPC and TFC, compared with the other investigated matrices. However, the ABTS response cannot be attributed exclusively to phenolic compounds, as other reducing or radical-scavenging constituents may also contribute to this chemical assay response. The higher ABTS values measured in the solvent-derived pomegranate peel extract may be related to the different extraction media and operating conditions used in the two procedures. The acidified methanol–water mixture and the pressurized water can provide different extraction responses depending on the characteristics of the matrix and the compounds present. In particular, the documented richness of pomegranate peel in phenolic constituents, including hydrolysable tannins and other polyphenols, may contribute to its high antioxidant response [43,44]. Nevertheless, because individual compounds were not characterized, it is not possible to establish whether the difference in ABTS response resulted from preferential recovery of specific phenolic compounds or from other extraction-dependent changes. All the other by-products showed substantially lower ABTS activity than pomegranate peel. Among these matrices, prickly pear peel extracts displayed the highest ABTS values, particularly when obtained by conventional solvent extraction. This result is consistent with the occurrence of phenolic compounds, betalains, and other antioxidant constituents reported for prickly pear tissues [42]. These compounds may contribute to the overall radical-scavenging response measured by ABTS [45]. The higher value observed for the conventionally extracted prickly pear samples may similarly reflect differences in the compounds recovered under the two extraction conditions. However, without individual compound characterization, the contribution of specific phenolic or non-phenolic constituents cannot be established. In contrast, turnip tops and artichoke stems and leaves showed lower ABTS responses than prickly pear residues. Although these by-products contain bioactive constituents with recognized health-promoting properties, their antioxidant capacity may be limited by the abundance and distribution of compounds contributing to the assay response or by the predominance of metabolites whose biological effects are not fully captured by the ABTS assay. For example, glucosinolate-derived compounds from turnip tops are more frequently associated with detoxification and chemoprotective mechanisms than with direct radical scavenging. Their health-promoting effects are mainly associated with the activation of endogenous antioxidant defence systems. Consequently, despite their well-established chemoprotective properties, glucosinolate-rich extracts often exhibit lower antioxidant capacity in chemical assays such as ABTS, which primarily measure the direct electron- or hydrogen-donating capacity of antioxidant molecules [46]. The lower ABTS values observed for these matrices should therefore not be interpreted as evidence of an absence of antioxidant compounds, but rather as a lower response under the specific chemical assay conditions used in the present study.

The results obtained with the FRAP assay, also reported in Table 2, confirmed the prominent antioxidant capacity of pomegranate peel extracts. Among all the analysed samples, pomegranate peel exhibited by far the highest ferric reducing antioxidant power, with the extract obtained by conventional solvent extraction showing significantly greater activity than the corresponding PLE extract (*p* < 0.05). This result is consistent with the substantially higher TPC and TFC values observed for pomegranate peel. Phenolic compounds can contribute to the FRAP response through their reducing capacity, although the assay response may also be affected by other reducing constituents present in the extracts [36]. In contrast to the pattern observed for pomegranate peel, all the other three by-products displayed an interesting extraction-dependent response in the FRAP values. The superior FRAP activity of solvent-derived pomegranate peel extract compared with the PLE extract is consistent with the higher TPC obtained using the acidified methanol–water system [36]. However, the present data do not allow the difference to be attributed to the recovery, degradation, or transformation of specific compounds. As discussed above, the two extraction procedures differed in solvent composition as well as in extraction conditions, and the observed difference may therefore result from their combined effects on the compounds contributing to the FRAP response. As regards the other matrices, the FRAP values were higher in the PLE extracts than in the corresponding conventionally extracted samples. This pattern was particularly evident for turnip tops and artichoke by-products, for which the PLE extracts showed approximately twice the FRAP response obtained with conventional extraction. This result indicates that the relative performance of the extraction procedures was assay- and matrix-dependent and that the higher TPC observed after conventional extraction did not necessarily correspond to a higher FRAP response for all matrices. As reported above, pressurized water at elevated temperature exerts various physicochemical properties. Such effects may differ according to matrix structure and composition and could contribute to the higher reducing capacity measured for the PLE extracts. However, because individual phytochemicals were not characterized, the present results do not allow the specific compounds responsible for this response to be identified, nor can specific extraction or transformation mechanisms be established.

The different responses observed between ABTS and FRAP demonstrate that antioxidant capacity cannot be described by a single chemical assay and that the response depends on both the matrix and the analytical endpoint. Turnip tops are mainly enriched in glucosinolates, whose biological activity is not related to direct electron donation. Artichoke residues predominantly contain chlorogenic acid and cynarin, which exhibit antioxidant properties but generally possess lower reducing capacity than the ellagitannins. Likewise, the antioxidant capacity of prickly pear residues is largely associated with betalains and ascorbic acid, compounds that may contribute less to ferric ion reduction than highly hydroxylated tannins [40,41,42].

To sum up, the results support the potential of pomegranate peel as an exceptionally rich source of bioactive compound, whereas the effect of extraction procedure varied according to both the matrix and the assay. Conventional extraction resulted in higher ABTS and FRAP responses for pomegranate peel, while PLE resulted in higher FRAP responses for the other three matrices. These findings further support the interpretation that extraction performance should be evaluated in relation to the specific matrix and analytical endpoint rather than considered as an intrinsic advantage of one extraction procedure over the other.

### 3.3. Results on Extract Cytotoxicity

**Figure 1:**
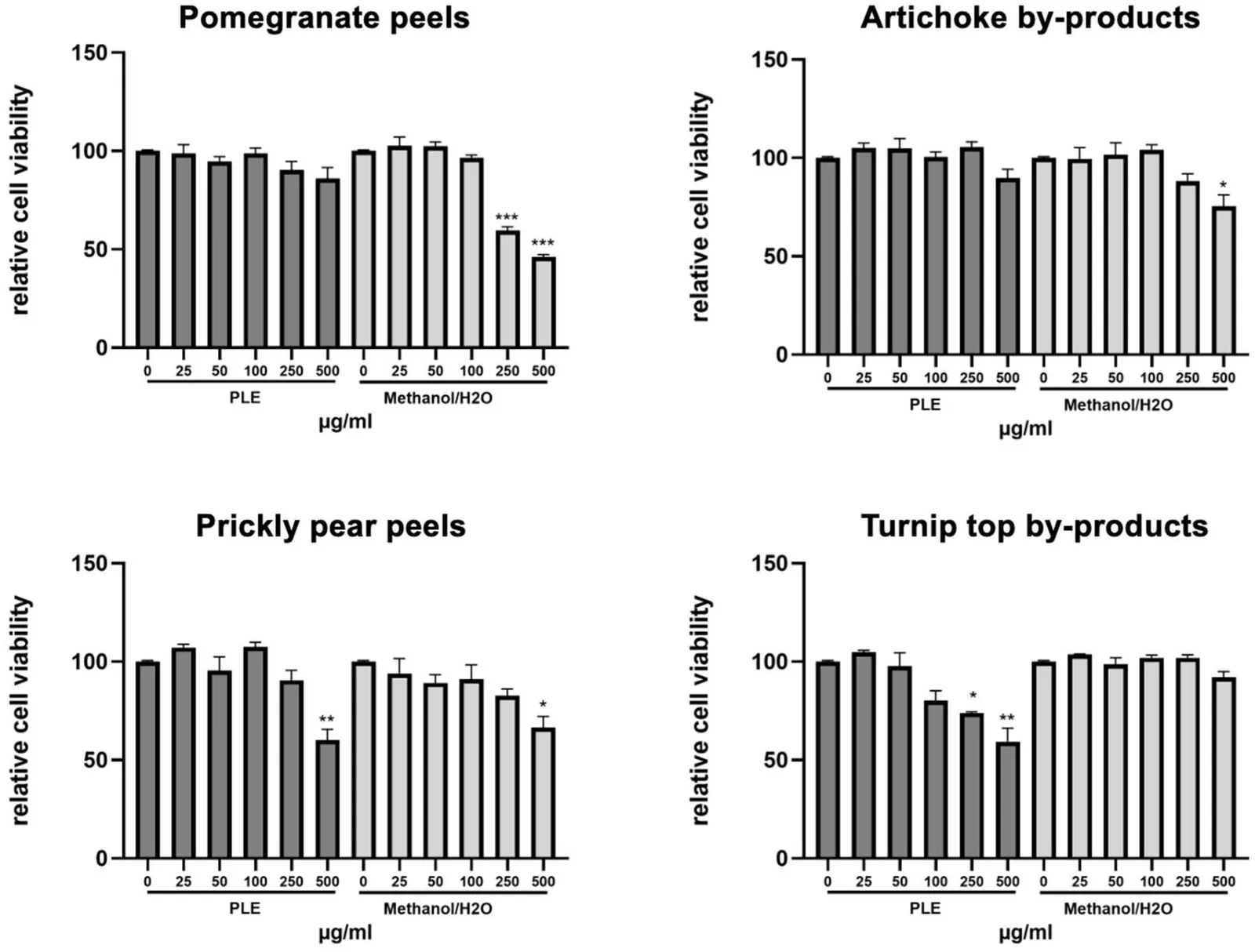
MTS assay of cell viability after 72 h exposure to increasing concentrations of the tested extracts. Data are presented as mean ± SD (n = 3). Statistical analysis was performed by one-way ANOVA followed by Dunnett’s post hoc test, *** *p* < 0.05, ** *p* < 0.01, * *p* < 0.001 vs. untreated control.

The cytotoxicity of the extracts was evaluated on Caco-2 cells after 72 h of exposure to concentrations ranging from 25 to 500 µg/mL using the MTS assay (Figure 1). Caco-2 cells are widely used as an in vitro model of the intestinal epithelium for the preliminary evaluation of the effects of food-derived bioactive compounds on cell viability and intestinal transport [47,48]. Despite the reduction in cell viability observed at the highest concentrations for some extracts, with values decreasing to approximately 50–60%, cell viability was largely preserved (generally remaining above 80%) at the lower concentrations tested, which represent the concentration range of greater interest for further investigation of the biological properties of these extracts. Similar findings were recently reported for other polyphenol-rich extracts obtained from agro-industrial by-products on the same cellular line, although after shorter exposure [49]. Based on the interpretation criteria commonly adopted in the literature [50], cell viability above 70% is generally considered indicative of the absence of relevant cytotoxicity. Overall, these findings indicate a concentration-dependent effect, with limited impact on cell viability at lower concentrations and more pronounced cytotoxic effects for some extracts at the highest concentrations tested. These findings support the further investigation of the extracts particularly within the lower concentration range associated with largely preserved cell viability and are consistent with previous reports describing the limited cytotoxicity of plant-derived polyphenol-rich extracts within concentration ranges commonly used for in vitro biological studies. Pomegranate peel and artichoke by-product extracts generally showed limited effects on Caco-2 viability at the lower concentrations tested. For pomegranate peel, a marked reduction in cell viability was observed only with the conventional methanol/H_2_O extract at the highest concentrations, whereas the PLE extract largely preserved cell viability across the tested concentration range. Artichoke extracts showed an overall limited effect on cell viability, with a reduction observed mainly at the highest concentration of the conventional methanol/H_2_O extract. The limited cytotoxicity of pomegranate peel extracts at lower concentrations and of the PLE-derived extract across the tested concentration range is consistent with previous reports describing the low cytotoxicity of polyphenol-rich pomegranate extracts despite their high hydrolysable tannin and phenolic content [39]. The reduction in cell viability observed at the highest concentrations may reflect the concentration-dependent behaviour of polyphenols, which at elevated concentrations have been reported to promote pro-oxidant mechanisms and impair mitochondrial function [30,36]. Similarly, the limited cytotoxicity of artichoke extracts agrees with previous reports describing extracts rich in caffeoylquinic acids as exhibiting low cytotoxicity in both non-cancerous and cancer cell models [51]. A slightly different behaviour was observed for turnip top extracts obtained by PLE, which showed a progressive decrease in Caco-2 viability at concentrations ≥ 250 μg mL^−1^. This response may be related to the characteristic phytochemical profile of Brassica vegetables. Turnip tops are particularly rich in glucosinolates, enzymatically converted into isothiocyanates upon tissue disruption. Although glucosinolates themselves are relatively inert, at high concentrations, isothiocyanates have been reported to affect cellular redox homeostasis and induce cell cycle arrest or apoptosis, which may contribute to the moderate reduction in cell viability observed in the present study [52]. Importantly, no relevant cytotoxicity was observed at lower concentrations, suggesting that the extract maintained limited effects on cell viability within the concentration range most likely to be relevant for potential nutraceutical applications. Prickly pear peel extracts also produced a moderate concentration-dependent decrease in cell viability for both extraction methods. Although *Opuntia ficus-indica* is generally considered a safe source of bioactive compounds, its extracts contain a complex mixture of betalains, phenolic acids, flavonoids, organic acids, and other secondary metabolites. Although these compounds are generally recognized for their antioxidant properties, their combined effects at higher concentrations may influence cellular metabolism and contribute to the modest reduction in cell viability observed in the present study [53].

To sum up, all investigated extracts had only limited effects on Caco-2 cell viability up to 100 μg mL^−1^, whereas concentration-dependent reductions in cell viability were observed only at the highest concentrations tested (250–500 μg mL^−1^). Notably, among the tested extracts, the PLE-derived pomegranate peel extract showed the most favorable combination of high antioxidant activity and low impact on cell viability, highlighting the potential of this extraction approach for obtaining bioactive extracts with a favorable in vitro profile. These findings support the further investigation of both conventional solvent- and PLE-derived extracts in relevant in vitro and in vivo models to better characterize their biological properties and evaluate their potential as functional ingredients.

## 4. Conclusions

This study provides a preliminary in vitro characterization of extracts obtained from four by-products using conventional solvent extraction and pressurized liquid extraction (PLE). The results showed that both the plant matrix and the extraction procedure influenced the phenolic and flavonoid contents and the chemical antioxidant capacity of the resulting extracts. Conventional extraction with acidified methanol–water generally resulted in higher Folin–Ciocalteu-estimated total phenolic contents and ABTS activity, whereas PLE produced comparable flavonoid contents and, for turnip tops and artichoke by-products, higher FRAP responses. These findings indicate that the relative performance of the two extraction procedures depends on both the characteristics of the matrix and the analytical endpoint considered.

Among the investigated matrices, pomegranate peel showed the highest total phenolic and flavonoid contents and the highest antioxidant capacity under both extraction conditions. However, the present results should not be interpreted as a comprehensive characterization of the phytochemical composition of the extracts, since individual compounds were not identified or quantified. Similarly, the differences observed between extraction procedures cannot be attributed to specific extraction, degradation, or transformation mechanisms on the basis of the present data alone. Rather, they may reflect the combined effects of solvent characteristics, extraction conditions, and matrix properties. All extracts showed low cytotoxicity toward Caco-2 cells under the tested experimental conditions, although some matrix- and concentration-dependent effects were observed. Further studies addressing individual phytochemical composition of the extracts using chromatographic approaches, bioaccessibility and bioavailability, stability during processing and storage, microbiological safety, and biological effects in appropriate models are warranted before potential food-related applications can be evaluated. From a process perspective, PLE enables the extraction step to be performed using water instead of an acidified organic solvent, thereby reducing the direct use of organic solvents during extraction. However, the present study did not quantitatively assess the overall environmental performance of the two procedures, and therefore no conclusion regarding their relative sustainability can be drawn. Optimization of PLE operating conditions may also help determine whether extraction performance can be improved for specific matrices and target responses.

## Figures and Tables

**Table 1:** Total polyphenol content (TPC) (mg GAE/g dw) and total flavonoid content (TFC) (mg QE/g dw) of the extracts from each by-product obtained by conventional solvent extraction (CSE) and by pressurized liquid extraction (PLE).

Extract	TPC [mg GAE/g dw]	TFC [mg QE/g dw]
Pomegranate peels—CSE	193.32 ± 0.26 ^a^	117.56 ± 0.92 ^a^
Turnip tops by-products—CSE	15.15 ± 0.67 ^d^	10.53 ± 0.19 ^c^
Prickly pear peels—CSE	14.59 ± 0.24 ^d^	5.82 ± 0.28 ^e^
Artichoke by-products—CSE	6.64 ± 0.61 ^f^	6.58 ± 0.57 ^d,e^
Pomegranate peels—PLE	141.72 ± 0.34 ^b^	90.68 ± 0.77 ^b^
Turnip tops by-product—PLE	17.01 ± 0.70 ^c^	11.40 ± 0.44 ^c^
Prickly pear peels—PLE	14.90 ± 0.56 ^d^	5.13 ± 0.10 ^e^
Artichokes by-product—PLE	10.23 ± 0.61 ^e^	7.86 ± 0.59 ^d^

Data are reported as means ± SD (*n* = 3). Experimental groups sharing different superscript letter are significantly different (*p* < 0.05).

**Table 2:** Antioxidant capacity by ABTS (mg Trolox/g dw) and FRAP (μmol Fe (II)/g dw) of the extracts from each by-product obtained by conventional solvent extraction (CSE) and by pressurized liquid extraction (PLE).

Extract	ABTS [mg Trolox/g dw]	FRAP [μmol Fe (II)/g dw]
Pomegranate peels—CSE	618.93 ± 13.93 ^a^	8015.41 ± 43.6 ^a^
Turnip tops by-products—CSE	22.90 ± 0.33 ^d,e^	145.03 ± 0.55 ^d^
Prickly pear peels—CSE	47.75 ± 0.36 ^c^	150.24 ± 0.55 ^d^
Artichoke by-products—CSE	9.78 ± 0.03 ^e^	81.25 ± 0.43 ^e^
Pomegranate peels—PLE	492.53 ± 15.13 ^b^	6032.21 ± 35.13 ^b^
Turnip tops by-product—PLE	23.53 ± 0.44 ^d,e^	295.91 ± 1.97 ^c^
Prickly pear peels—PLE	34.96 ± 0.74 ^c,d^	190.18 ± 0.64 ^d^
Artichokes by-product—PLE	11.54 ± 0.64 ^e^	145.69 ± 0.44 ^d^

Data are reported as means ± SD (*n* =3). Experimental groups sharing different superscript letter are significantly different (*p* < 0.05).

## Data Availability

The original contributions presented in the study are included in the article, further inquiries can be directed to the corresponding authors.
